# Comparative In Vitro Analysis of Fracture Toughness and Flexural Strength in Interim Fixed Partial Dentures Reinforced With Various Nanofibers

**DOI:** 10.7759/cureus.76069

**Published:** 2024-12-20

**Authors:** Thimmappa Meenakshi, Sneha B Kolla, Bhoomika B M, Kolla Jaswanth

**Affiliations:** 1 Prosthodontics and Crown and Bridge, The Oxford Dental College, Bengaluru, IND

**Keywords:** carbon fibres, fixed partial dentures, glass fibres, kevlar fibres, polymethyl methacrylate, reinforced pmma

## Abstract

Aim

To evaluate and compare the fracture toughness and flexural strength of interim fixed partial dentures reinforced with carbon, glass, and Kevlar nanofibers.

Materials and methods

This study explored the effect of reinforcing poly methyl methacrylate with carbon, glass, and Kevlar fibers on its fracture toughness and flexural strength. A total of 120 samples were prepared, divided into two groups of 60 samples each, with one group tested for fracture toughness and the other for flexural strength. Each group of 60 samples was further subdivided into four equal subgroups, consisting of carbon, glass, Kevlar, and control groups (no reinforcement). The samples were tested using a universal testing machine, and the results were analyzed statistically.

Results

The normality of the continuous data was assessed using the Kolmogorov-Smirnov and Shapiro-Wilk tests. Continuous data were presented as mean and standard deviation. ANOVA was employed to determine significant differences in means across the groups, and the Post Hoc Bonferroni test was used for intergroup comparisons. A p-value of less than 0.05 was considered statistically significant. Kevlar fibers showed the highest mean value for fracture toughness (734.81 ± 58.22 MPa·m1/2), while glass fibers had the highest mean value for flexural strength (1.20 ± 0.05 MPa).

Conclusion

Kevlar fibers demonstrated greater fracture toughness compared to glass and carbon fibers. In terms of flexural strength, glass fibers exhibited the highest values, followed by Kevlar, with carbon fibers showing the lowest. The group without any reinforcement had the lowest values for both fracture toughness and flexural strength.

## Introduction

A provisional restoration is an important phase in fixed prosthodontic therapy. The word 'provisional' means established for the time being. It should have good marginal integrity and aesthetics, protect the pulpal and periodontal tissues, and be durable enough to endure the stresses of mastication [1]. For patients with a treatment plan that requires long-term use of provisional restorations, like full mouth rehabilitation [2], materials with improved mechanical properties are required. The interim coverage is especially critical for patients in cases where fixed restorative therapy is to be given along with periodontal therapy or full-mouth rehabilitation is to be done [3]. Polymers such as polymethyl methacrylate (PMMA), polyethyl methacrylate (PEMA), and polybutyl methacrylate (PBMA) are commonly used as provisional restorative materials. These materials typically consist of a polymer powder and a monomer liquid system. Additionally, polymers like dimethacrylates, urethane dimethacrylates, and epimines are employed in interim restorations [4]. Owing to its excellent qualities, PMMA has become the most commonly used provisional restorative material in prosthodontics. However, research has shown that PMMA has certain shortcomings in its mechanical and physical properties [5]. To overcome the limitations of PMMA, various reinforcing fibers such as glass, carbon, Kevlar, and high-molecular-weight polyethylene have been used as reinforcement materials [5]. Kevlar fiber (para-aramid) is popular as it exhibits superior mechanical properties compared to nylon and glass fibers, has superior wettability compared to carbon fibers, and does not require treatment with a coupling agent. Kevlar fibers have been found to enhance tensile strength, elastic modulus, impact resistance, and fracture toughness while being biocompatible and non-toxic [6]. Glass fibers have gained significant attention in dentistry due to their aesthetic appeal, flexibility, and biocompatibility. Glass-fiber-reinforced specimens have demonstrated the highest flexural strength compared to other fibers [7]. Carbon fibers, known for their high strength-to-weight ratio, enhance both the tensile and flexural strength of PMMA when incorporated. Their stiffness also improves the rigidity of PMMA composites [8].

Previous studies have highlighted the potential of fiber reinforcements in improving the mechanical performance of dental materials. Carbon fibers, with their strength and stiffness, are ideal for enhancing load-bearing capacity [9], while glass fibers are appreciated for their biocompatibility and ease of integration into dental composites [10]. Kevlar fibers offer excellent impact resistance and flexibility, making them uniquely suited for dental applications [11].

A research gap was identified in the need for a comparative analysis of these fibers' performance in interim fixed partial dentures. This led to the formulation of a research question aimed at evaluating and comparing the fracture toughness and flexural strength of interim fixed partial dentures reinforced with glass, Kevlar, and carbon fibers. The primary objective of this study was to assess the fracture toughness and flexural strength of interim fixed partial dentures reinforced with different fibers. The secondary objective was to determine which of the three materials is most suitable for interim restorations. The null hypothesis proposed that reinforcing PMMA with fibers would not result in any significant difference in its fracture toughness and flexural strength.

## Materials and methods

The ethical clearance for the research work was obtained from the Institutional Ethics Committee (reference number TODC/034/ECAL/2021-22). The sample size was determined based on prior studies assessing fracture toughness and flexural strength in fiber-reinforced interim fixed partial dentures. Statistical power analysis was used to ensure reliable comparisons among glass, carbon, and Kevlar reinforcements, resulting in a minimum requirement of 15 samples per group to achieve a 5% significance level.

**Figure 1:**
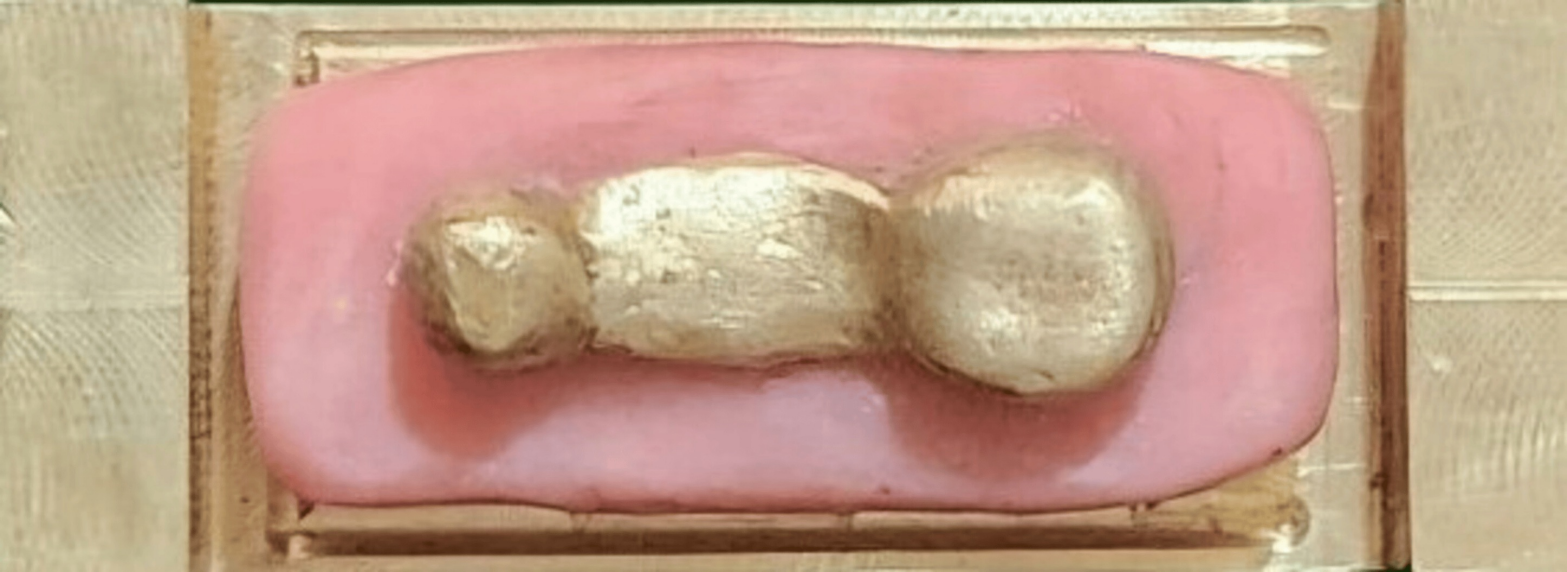
Master die.

**Figure 2:**
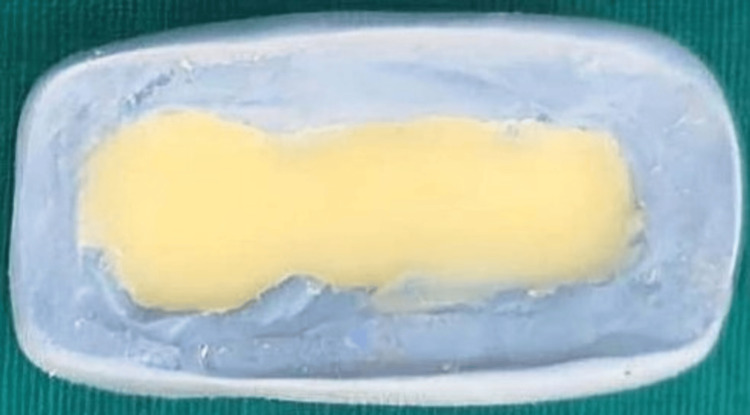
Putty index with resin.

**Figure 3:**
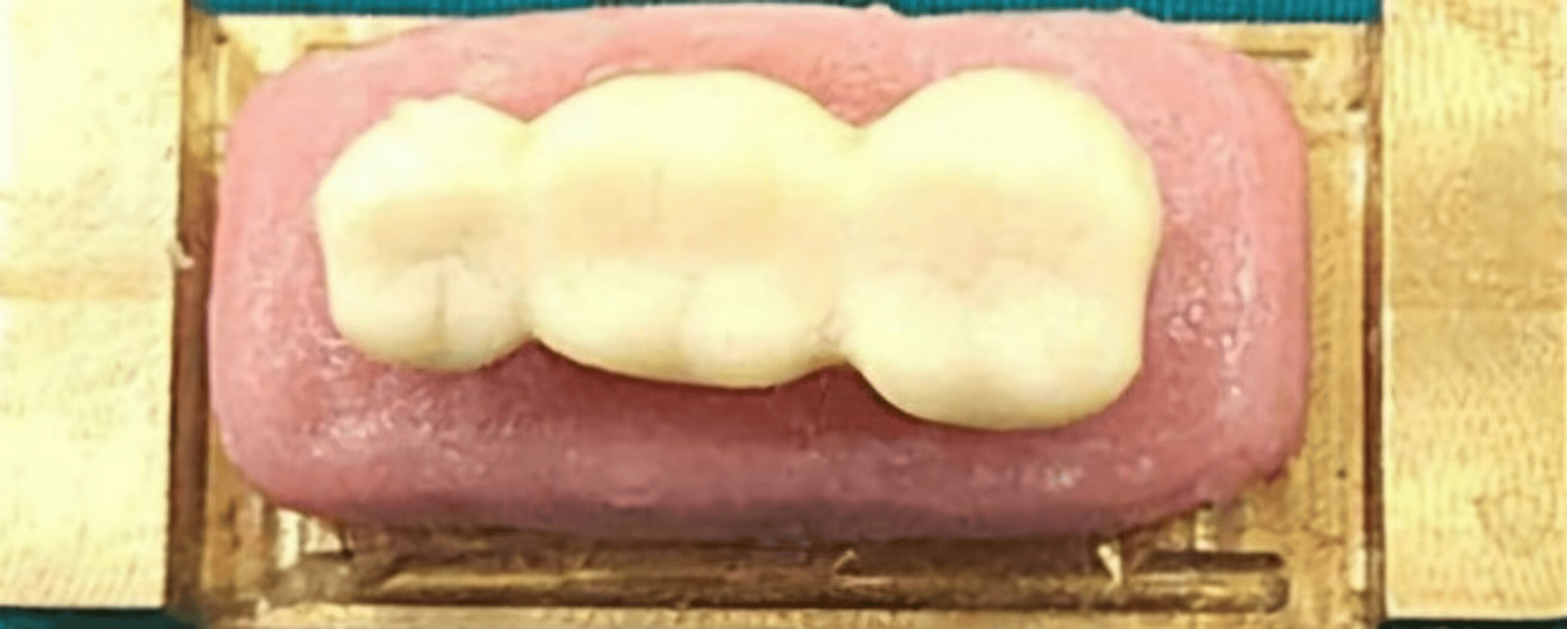
Prepared sample.

Tooth preparation of the 45 and 47 was performed on a typodont model (Nissin, Japan). An impression of the prepared teeth was made using polyvinyl siloxane impression material (GC Flexceed, Japan) with a perforated sectional impression tray. The impression was scanned using an extraoral scanner (Cyber-scan, UK). The space between the abutments was cast to simulate the edentulous ridge with an interabutment distance of 10 mm. The mesiodistal and buccolingual widths of the connector were kept at 3.5 mm, and the occluso-cervical length was 2.5 mm. A computer-aided design/computer-aided manufacturing (CAD/CAM system) (AutoCAD software, Medit 500) milled a brass master die and a 3-unit chrome cobalt metal bridge. The master die was secured to a brass metal base with auto-polymerizing resin (Figure 1). A 3-unit full metal prosthesis was secured on the master die, and a putty index of the prosthesis was made using polyvinyl siloxane impression material. To create the interim prosthesis, auto-polymerizing acrylic resin was mixed according to the manufacturer’s instructions in a dappen dish with a cement mixing spatula. The mixed resin was then placed into the putty index. Precut fibers (glass, Kevlar, carbon), 1 mm thick and 10 mm long, were soaked in methyl methacrylate monomer for 10 seconds and positioned in the pontic area of the index, spanning between the connectors (Figure 2). The filled putty index was carefully aligned and placed onto the die, ensuring accurate positioning. A rubber band was used to apply uniform pressure to the index, and the material was allowed to polymerize for 15 minutes. Once polymerization was complete, the interim prosthesis was removed, trimmed, and polished (Figure 3).

Inclusion and exclusion criteria for samples

Samples with no occlusal or marginal discrepancies and free from defects were included in the study. Materials showing any of these issues were excluded, and new samples were fabricated in their place.

**Figure 4:**
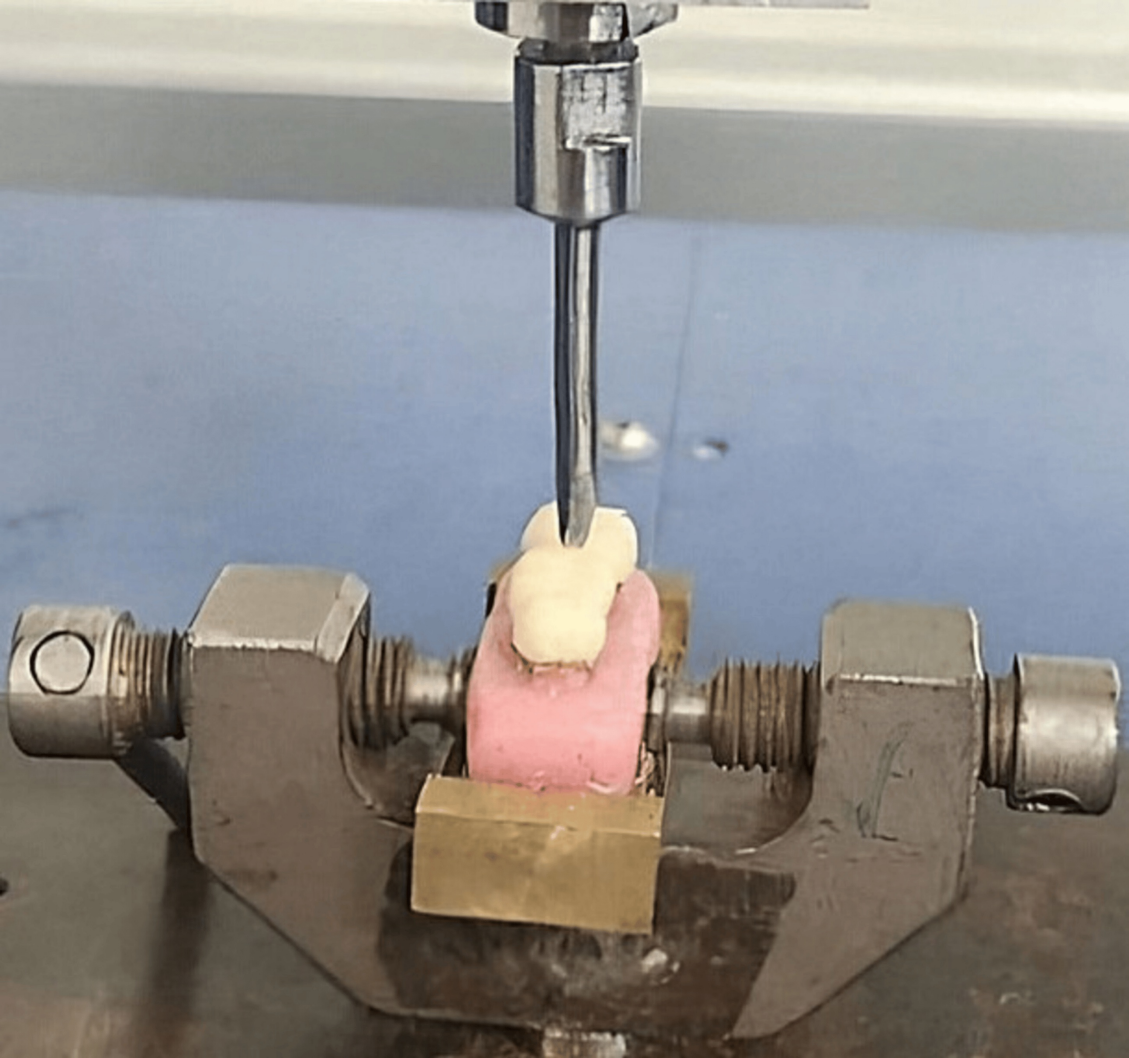
Testing for fracture toughness.

In total, 120 samples were produced. These samples were divided into two groups: sixty samples were tested for fracture toughness, and the remaining sixty for flexural strength. Each group was further divided into four subgroups of 15 samples each, consisting of glass, Kevlar, and carbon fibers, with one subgroup having no fiber reinforcement. Fracture toughness was evaluated using a universal testing machine (Mecmesin, Multitest 10i, Japan), with the force applied perpendicular to the occlusal plane of each sample. The sample was secured in a specially designed tension device (Figure 4) within the machine, and a tensile force was applied at a crosshead speed of 5 mm per minute; the load at crack initiation was recorded. The peak force (F), measured in Newtons, which led to the fracture of the samples, was recorded and used to calculate the fracture toughness (Kc) in MPa·m1/2 using Srawley’s equation (1976): Kc = (Pc/Bw1/2) * F(a/w), where Pc is the load at crack initiation in Newtons, B is the thickness of the sample in centimeters, w is the width of the sample in centimeters, and a is the crack length in centimeters.

**Figure 5:**
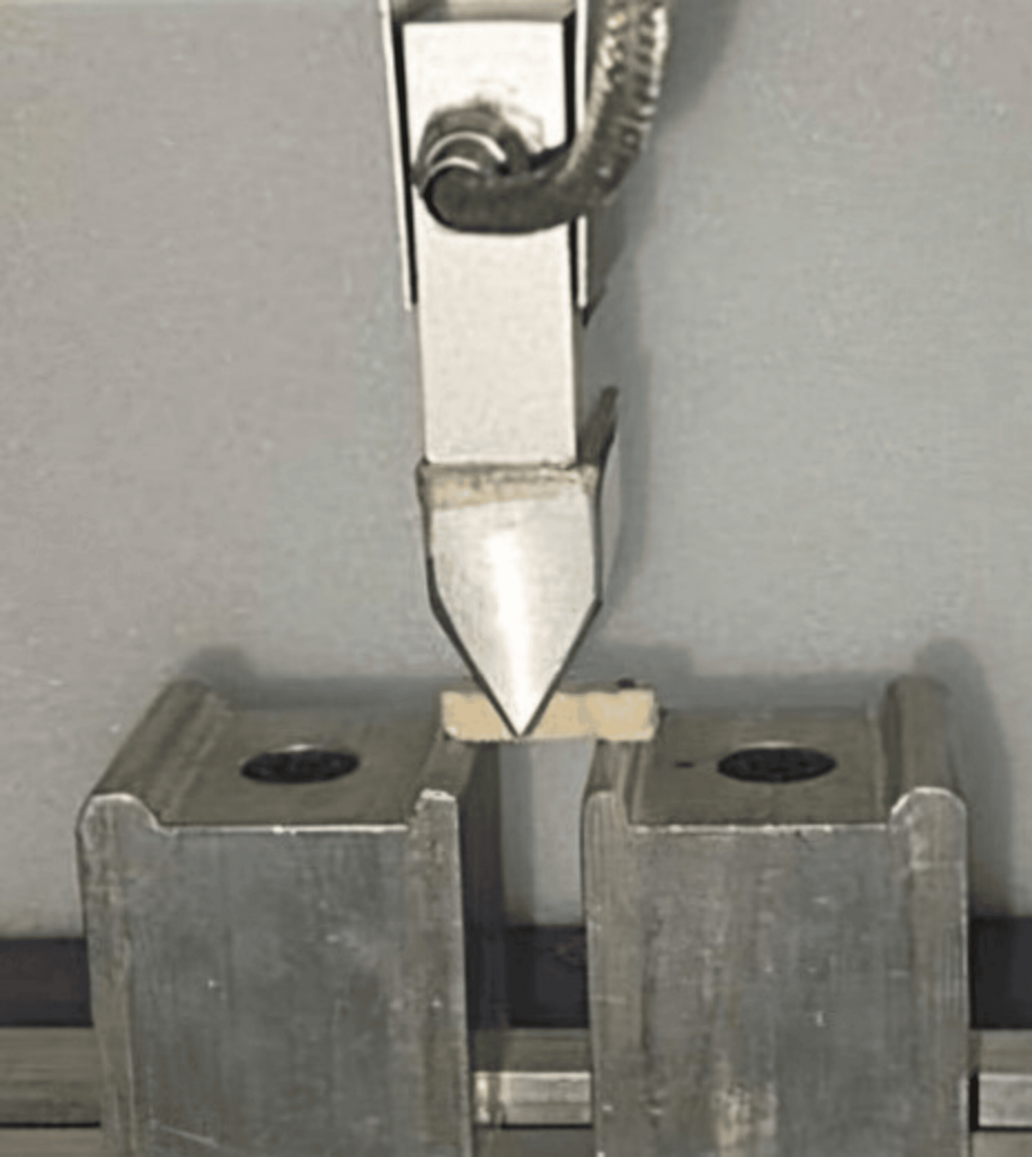
Testing for flexural strength.

Flexural strength was measured using a universal testing machine. Each sample was positioned on a bending fixture with two parallel supports, each 2 mm in diameter and spaced 20 mm apart. A load was applied at a crosshead speed of 2 mm per minute using a 2-mm rod placed centrally between the supports. The peak force (F) in Newtons, obtained from the stress-strain curve of each specimen (Figure 5), was recorded and used to calculate the flexural strength in MPa using the following equation: δβ = 3FI/2bh2, where δβ is the flexural strength in MPa, F is the maximum fracture load in Newtons, I is the distance between the supports in millimeters, b is the breadth of the test specimens in millimeters, and h is the height of the test sample in millimeters.

The results obtained were subjected to statistical analysis. Categorical data was presented as frequencies and proportions. The normality of continuous data was assessed using the Kolmogorov-Smirnov and Shapiro-Wilk tests. Continuous data were expressed as mean and standard deviation. ANOVA was used to test for significant differences in means between more than two groups for quantitative data. Intergroup analysis was performed using the Post Hoc Bonferroni test. A significance level of 5% was applied.

## Results

The fracture toughness and flexural strength of the samples were assessed and compared across four subgroups: one without reinforcement, one reinforced with glass fibers, one with Kevlar fibers, and one with carbon fibers. The mean fracture toughness and flexural strength values were calculated using the SPSS statistical software package (version 20, IBM Corp., released 2011). ANOVA results showed a significant difference (p < 0.05) in fracture toughness among the groups. The detailed analysis is presented in Tables 1-3. Table 1 exhibits the mean, standard deviation, and median values, revealing that the Kevlar fiber group had the highest mean peak force of 668.81 N. Table 2 shows the relative fracture toughness of each group compared to the overall toughness observed. Table 3 provides a comparative analysis of fracture toughness across the groups, showing significant differences, with Kevlar fiber showing notably higher fracture toughness.

**Table 1:** The peak force (N) required for fracture toughness.

Subjects (N=60)	Mean	SD	Median
Control group (N=15)	126.52	3.35	125.60
Glass Fibre (N=15)	333.25	48.09	324.47
Kevlar Fibre (N=15)	668.81	31.38	674.30
Carbon Fibre (N=15)	224.85	19.77	214.60

**Table 2:** Fracture toughness values.

Subjects (N=60)	Mean	SD	Median
Control group (N=15)	112.56	8.20	112.50
Glass Fibre (N=15)	420.66	51.73	444.85
Kevlar Fibre (N=15)	734.81	58.22	717.58
Carbon Fibre (N=15)	253.99	45.44	250.37

**Table 3:** Comparison of fracture toughness between the groups. # Analysis of variance * Statistically significant

Fracture Toughness	Sum of Squares	Degree of freedom	Mean Square	F-value	p-value^#^
Between Groups	1.994	3	0.665	219.815	<0.001*
Within Groups	0.169	56	0.003	-	-
Total	2.163	59	-	-	-

Analysis of flexural strength is shown in Tables 4-6. In Table 4, the glass fiber group showed the highest mean peak force of 126.30 N. The flexural strength was assessed within each group and depicted in Table 5. The glass fiber exhibited the highest flexural strength, constituting the maximum with a mean value of 1.20 MPa. Table 6 depicts the comparative analysis of the flexural strength across the distinct material groups. Notably, glass fiber material demonstrated substantially elevated flexural strength compared to Kevlar fiber and carbon fiber.

**Table 4:** The peak force (N) required flexural strength.

Subjects (N=60)	Mean	SD	Median
Control group (N=15)	73.09	3.69	72.98
Glass Fibre (N=15)	126.30	3.93	125.65
Kevlar Fibre (N=15)	94.17	4.19	95.48
Carbon Fibre (N=15)	80.72	0.43	80.74

**Table 5:** Flexural strength values.

Subjects (N=60)	Mean	SD	Median
Control group (N=15)	0.69	0.05	0.69
Glass Fibre (N=15)	1.20	0.05	1.20
Kevlar Fibre (N=15)	0.94	0.07	0.95
Carbon Fibre (N=15)	0.87	0.05	0.87

**Table 6:** Comparison of flexural strength between the groups. # Analysis of variance * Statistically significant

Flexural Strength	Sum of Squares	Degree of freedom	Mean Square	F-value	p-value^#^
Between Groups	24838.628	3	8279.543	707.754	<0.001*
Within Groups	655.106	56	11.698	-	-
Total	25493.734	59	-	-	-

## Discussion

Carbon fibers are known for their high tensile strength, low weight, and resistance to corrosion and fatigue. By embedding these fibers within the PMMA matrix, the resulting material can exhibit increased tensile strength, improved impact resistance, and reduced weight compared to pure PMMA [9]. Adding glass fibers to PMMA resin can enhance its mechanical properties, including strength and durability, making it suitable for use in interim FPDs. The addition of glass fibers helps to reinforce the PMMA material, reducing the risk of fracture and improving its overall performance as an interim dental restoration [10]. Kevlar fibers are popular as they exhibit superior mechanical properties compared to nylon and glass fibers. Polyaramid fibers have superior wettability compared to carbon fibers and do not require treatment with a coupling agent [11]. The present study aimed to evaluate the effect of glass, Kevlar, and carbon fiber reinforcement on fracture toughness and flexural strength of PMMA provisional restorative material. It also intended to compare the three materials on fracture toughness and flexural strength without any fiber reinforcement. The null hypothesis formulated was rejected. The stress at which a brittle material fractures is called the fracture strength. In the present study, samples were tested in tension in a universal testing machine with the force direction perpendicular to the plane of the preformed crack. Flexural strength, also known as bending strength or modulus of rupture, is a mechanical property of a material that measures its ability to withstand bending without breaking. Each sample was held in a specially designed tension device in the machine, and a tension force was applied at a crosshead speed of 5 mm/min for fracture toughness and 2 mm/min for flexural strength.

Table 1 presents the mean values of force (N) required to fracture the samples, which were used to determine fracture toughness. The Kevlar fiber group showed the highest mean peak force at 668.81 ± 31.38 N, followed by the glass fiber group at 333.25 ± 48.09 N and the carbon fiber group at 224.85 ± 19.77 N. In contrast, the control group exhibited the lowest mean peak force, 126.52 ± 3.35 N. Table 2 shows the mean, standard deviations, and median values for fracture toughness. The Kevlar fiber group recorded the highest mean peak force at 734.81 ± 58.22 MPa.m1/2, followed by the glass fiber group at 420.66 ± 51.73 MPa.m1/2, and the carbon fiber group at 253.99 ± 45.44 MPa.m1/2. The control group displayed the lowest mean peak force at 112.56 ± 8.20 MPa.m1/2. Table 3 represents a comparative analysis of the fracture toughness of interim fixed partial dentures across different groups, revealing a statistically significant difference among them. The results indicate that Kevlar fiber stands out as the superior material, displaying notably higher fracture toughness compared to alternative materials like glass fiber and carbon fiber. Furthermore, the absence of any fiber in the composition resulted in the lowest observed fracture toughness.

Kevlar fiber exhibits high fracture toughness primarily due to its unique molecular structure and mechanical properties. Kevlar is a synthetic fiber made from aromatic polyamides. These fibers exhibit excellent thermal stability over a wide range of temperatures. They maintain their mechanical properties even at elevated temperatures, which contributes to their high fracture toughness in various operating conditions [12].

Table 4 presents the mean values of force (N) required to fracture the samples to determine flexural strength. The glass fiber group exhibited the highest mean ± standard deviation at 126.30 ± 3.93 N, followed by the Kevlar fiber group at 94.17 ± 4.19 N, and the carbon fiber group at 80.72 ± 0.43 N. The control group recorded the lowest mean flexural strength at 73.09 ± 3.69 N. Table 5 displays the mean, standard deviation, and median values for flexural strength. The glass fiber group had the highest mean ± standard deviation for fracture toughness at 1.20 ± 0.05 MPa, followed by the Kevlar fiber group at 0.94 ± 0.07 MPa, and the carbon fiber group at 0.87 ± 0.05 MPa. In contrast, the control group showed the lowest mean fracture toughness at 0.69 ± 0.05. Table 6 represents the comparison of flexural strength of interim fixed partial dentures in each group, and the study found statistically significant differences between the groups. The findings suggest that glass fiber emerges as the optimal material, demonstrating markedly higher flexural strength in contrast to alternative materials such as Kevlar fiber and carbon fiber. Additionally, the absence of any fiber in the composition resulted in the lowest observed flexural strength.

Glass fibers exhibit high flexural strength primarily due to their composition, manufacturing process, and inherent properties. The composition of glass fibers includes silica (SiO2) as the primary component, along with various additives to enhance properties such as flexibility and durability. Glass fibers can be oriented in a specific direction during the manufacturing process, which enhances their strength in that direction [5-11].

An in-vitro study by Kapri [11] in 2014 evaluated the fracture load values of PMMA reinforced with glass fibers at three different locations mainly occlusal, cervical, and middle. The results showed that the placement of the reinforcement in the occlusal third of the pontic resulted in higher fracture resistance which was significantly higher (P < 0.05) than all other locations. In 2020, Gopinath A et al. [6] compared the fracture toughness and flexural strength of PMMA and bisacryl composite provisional restorative materials with and without reinforcement of Kevlar fibers. The mean fracture toughness and flexural strength values obtained were significantly higher for the bisacryl composite when compared to PMMA.

A comparison of flexural strength and hardness of heat-cure PMMA, when reinforced with Kevlar fibers, nylon fibers, metal mesh, and fiberglass mesh, was done in 2023 by Parveen SR et al. [12]. Specimens reinforced with glass fiber mesh showed the highest flexural strength, followed by metal mesh. The hardness of Kevlar fibers decreased slightly compared to the control group. John J et al. [13] in 2001 performed an in-vitro study to determine whether heat-polymerized acrylic denture base material could be improved through reinforcement with glass, aramid, or nylon fibers. Specimens reinforced with glass fibers showed the highest flexural strength, followed by aramid and nylon.

Limitations

This study was an in-vitro study and cannot be directly compared to clinical situations because of the biological variables of the oral cavity. No cyclic loading in a moist environment was performed in the present study, which may affect the application of the results to real-world scenarios.

Scope for future research

Future research could focus on optimizing fiber reinforcements by exploring different combinations, orientations, and lengths to enhance the fracture toughness and flexural strength of interim fixed partial dentures. Additionally, randomized clinical control studies involving PMMA reinforced with different nano fibers can be considered to predict long-term durability along with the assessment of biocompatibility.

## Conclusions

In conclusion, this research work presents a thorough evaluation and comparative analysis of the fracture toughness and flexural strength of interim fixed partial dentures reinforced with three different types of nanofibers. The control group demonstrated the lowest fracture toughness and flexural strength values when compared to the nanofiber-reinforced groups. This indicates that the reinforcement of provisional restorative materials is necessary to enhance their mechanical durability and fracture resistance, thereby improving their longevity. Kevlar fibers demonstrated the highest fracture toughness among the tested groups, while glass fibers exhibited the greatest flexural strength. In contrast, carbon fibers recorded the lowest values for both properties.
